# Molecular epidemiology of drug-resistant *Neisseria gonorrhoeae* in Russia (Current Status, 2015)

**DOI:** 10.1186/s12879-016-1688-7

**Published:** 2016-08-09

**Authors:** Alexey Kubanov, Denis Vorobyev, Aleksandr Chestkov, Arvo Leinsoo, Boris Shaskolskiy, Ekaterina Dementieva, Viktoria Solomka, Xenia Plakhova, Dmitry Gryadunov, Dmitriy Deryabin

**Affiliations:** 1 State Research Center of Dermatovenerology and Cosmetology, Korolenko str. 3/1, Moscow, 107076 Russia; 2 Engelhardt Institute of Molecular Biology, Russian Academy of Sciences, 32 Vavilov str., Moscow, 119991 Russia

**Keywords:** *Neisseria gonorrhoeae*, Russia, Epidemiology, NG-MAST, Antimicrobial resistance, Genetic determinants of drug resistance

## Abstract

**Background:**

The widespread distribution of *Neisseria gonorrhoeae* strains that are resistant to previously used and clinically implemented antibiotics is a significant global public health problem. In line with WHO standards, the national Gonococcal Antimicrobial Surveillance Programme (RU-GASP) has been in existence in Russia since 2004; herein, the current status (2015) is described, including associations between *N. gonorrhoeae* antimicrobial susceptibility, primary genetic resistance determinants and specific strain sequence types.

**Methods:**

A total of 124 *N. gonorrhoeae* strains obtained from 9 regions in Russia in 2015 were examined using *N. gonorrhoeae* Multi-Antigen Sequence Typing (NG-MAST), an antimicrobial susceptibility test according to European Committee on Antimicrobial Susceptibility Testing (EUCAST) criteria and an oligonucleotide microarray for the identification of mutations in the *penA*, *ponA*, *rpsJ*, *gyrA* and *parC* genes responsible for penicillin G, tetracycline, and fluoroquinolone resistance. Genogroup (G) isolates were evaluated based on their *porB* and *tbpB* sequence types (STs).

**Results:**

NG-MAST analysis showed a diversified population of *N. gonorrhoeae* in Russia with 58 sequence types, 35 of which were described for the first time. The STs 807, 1544, 1993, 5714, 9476 and 12531, which were typical for some Russian Federation regions and several countries of the former Soviet Union, were represented by five or more isolates. The internationally widespread ST 1407 was represented by a single strain in the present study. Division into genogroups facilitated an exploration of the associations between *N. gonorrhoeae* sequence type, antimicrobial resistance spectra and genetic resistance determinant contents. Preliminarily susceptible (G-807, G-12531) and resistant (G-5714, G-9476) genogroups were revealed. The variability in the most frequently observed STs and genogroups in each participating region indicated geographically restricted antimicrobial susceptibility in *N. gonorrhoeae* populations.

**Conclusions:**

Resistance or intermediate susceptibility to previously recommended antimicrobials, such as penicillin G (60.5 %), ciprofloxacin (41.1 %) and tetracycline (25 %), is common in the *N. gonorrhoeae* population. Based on previous reports and current data, ceftriaxone and spectinomycin should be recommended for first-line empiric antimicrobial monotherapy for gonorrhoea in Russia.

**Electronic supplementary material:**

The online version of this article (doi:10.1186/s12879-016-1688-7) contains supplementary material, which is available to authorized users.

## Background

Infections caused by *Neisseria gonorrhoeae* are significant public health problems globally [1], in EU counties [2, 3], and in the former Soviet Union states [2, 4, 5]. The incidence of gonorrhoea in Russia (146 million inhabitants) has declined in the past 15 years; however, the rate of 23.5 reported cases per 100,000 members of the population is very high for the WHO European Region [http://data.euro.who.int/cisid/Default.aspx?TabID=394725].

This phenomenon presents with increased urgency because *N. gonorrhoeae* has developed resistance to antimicrobial drugs that were previously and are currently recommended for gonorrhoea treatment [6]. The resistance of *N. gonorrhoeae* to penicillin G is associated with mutations in the chromosomal *ponA* and *penA* genes, which decrease the affinity of the corresponding penicillin-binding proteins PBP1 and PBP2 [7, 8]. For example, an aspartate insertion at amino acid position 345 of PBP2 indicates a significant mutation towards intermediate susceptibility or resistance to penicillin G [8, 9]. The major determinant of tetracycline resistance includes mutations in the *rpsJ* gene, which results in the amino acid substitution V57M or V57L in the ribosomal protein S10 [6, 10]. Resistance to fluoroquinolones is caused by mutations in *gyrA* and *parC* genes encoding the gyrase and topoisomerase polypeptide chains that form the “quinolone pocket”. Isolates with high-level fluoroquinolone resistance may carry several mutations in *gyrA* at positions 91 and 95 and in *parC* at positions 87 and 91 [11]. Numerous other chromosomal and plasmid drug resistance determinants have been described in clinical *N. gonorrhoeae* isolates worldwide [6] following the introduction of antimicrobials into clinical practice.

In response to this emerging situation, the WHO [12] and the European Centre for Disease Prevention and Control [13] published global and region-specific response plans to mitigate and control the spread of drug-resistant *N. gonorrhoeae*. In Russia, the national Gonococcal Antimicrobial Surveillance Programme (RU-GASP) was initiated in 2004 in accordance with WHO standards.

A key component in these plans and programmes is to use a number of rapid and sensitive methods to characterise both the genotypes and drug resistance phenotypes of *N. gonorrhoeae* strains. For molecular epidemiological typing, the *N. gonorrhoeae* Multi-Antigen Sequence Typing (NG-MAST) method, which differentiates strains on the basis of sequence variation in two hypervariable *porB* and *tbpB* loci, has been used in many countries worldwide [14–16]. In turn, antimicrobial resistance is traditionally determined using the agar dilution method according to the recommendations from the US Clinical and Laboratory Standards Institute [17]. In 2010–2013, this approach successfully identified associations between NG-MAST and antimicrobial resistance profiles to determine the ability of spreading drug-resistant strains in Poland [3], Belorussia [5] and Russia [4]. However, the most recent study on isolates from Russia was internationally published in 2014, and there are limited data regarding drug resistance determinants in circulating strains; genetic techniques are increasingly used for this purpose [18, 19].

The aims of this study were to investigate the current (2015) molecular epidemiology of *Neisseria gonorrhoeae* isolates from Russia using the NG-MAST method and to determine the associations between antimicrobial susceptibility, the main genetic resistance determinants and specific strain sequence types.

## Methods

### Collection and identification of *Neisseria gonorrhoeae* isolates

The present study was conducted according to the Russian Gonococcal Antimicrobial Surveillance Programme (RU-GASP) in 9 participating regions. The study duration was from January to December 2015. According to the Russian Ministry of Health national guidelines № 1177n each patient signed the informed consent for use of clinical specimen in RU-GASP. Urethral and cervical specimens from females and urethral specimens from males were collected and cultured on chocolate blood agar supplemented with 1 % IsoVitaleX enrichment and 1 % VCAT (vancomycin, colistin, amphotericin and trimethoprim) selective supplement (Becton Dickinson, Sparks, MD, USA). The primary species identification of *N. gonorrhoeae* was performed based on Gram staining and rapid oxidase reaction in participating regions, after which the frozen samples were transported to the State Research Center of Dermatovenerology and Cosmetology (Moscow). Finally, all strains were verified via a sugar utilisation test performed with an NH ID card for the VITEK 2 Compact analyser (bioMérieux, Marcy l’Etoile, France) and via MALDI-TOF MS using the MALDI Biotyper (Bruker Daltonics, Bremen,Germany). The 124 *N. gonorrhoeae* isolates from females (*n* = 32) and males (*n* = 92) were included in these research, preserved in cryomedium at -70 °C and then used for DNA extraction and antimicrobial sensitivity tests.

### *Neisseria gonorrhoeae* genomic DNA isolation and molecular typing (NG-MAST) protocol

DNA samples for each isolate were prepared from several *N. gonorrhoeae* suspensions using the DNA-Express kit (Lytech, Moscow, Russia) according to the manufacturer’s instructions and stored at -20 °C for subsequent use.

For molecular typing, the NG-MAST protocol was performed [20]. More variable internal regions of the *porB* and *tbpB* genes were amplified via PCR, and the subsequent products were purified and sequenced using a 3730xl Genetic Analyser (Applied Biosystems, Forster City, CA, USA). Both the leading and reverse strands were assessed; the sequencing data were processed using 3730/3730xl Data Collection Software v3.0. Allele numbers for the *porB* (490 bp) and *tbpB* (390 bp) sequences and sequence types were assigned via the online NG-MAST database (www.ng-mast.net). To define the *N. gonorrhoeae* genogroups, the similarities of the *porB* and *tbpB* alleles were determined using the maximum likelihood test and edited using Molecular Evolutionary Genetics Analysis version 6 (MEGA6) software [21].

### *Neisseria gonorrhoeae* antimicrobial susceptibility testing

The minimum inhibitory concentrations (MICs; mg/L) of penicillin G, ceftriaxone, tetracycline, spectinomycin, azithromycin and ciprofloxacin were analysed via the MIC method using agar dilution methodology on chocolate blood agar enriched with 1 % IsoVitaleX (Becton Dickinson, Sparks, MD, USA) according to the recommendations of the US Clinical and Laboratory Standards Institute [17].

For quality control, the *N. gonorrhoeae* ATCC 49226 reference strain was included twice in each test. All results were interpreted using whole MIC dilutions. Where available, breakpoints for susceptibility (S), intermediate susceptibility (I) and resistance (R) according to The European Committee on Antimicrobial Susceptibility Testing (EUCAST; www.eucast.org) were used (Table 1).

**Table 1:** Antimicrobial susceptibility of 124 *Neisseria gonorrhoeae* isolates from Russia in 2015

Antimicrobials (breakpoints; mg/L) ^a^	Number (%) of isolates			
	Susceptible (S)	Intermediate susceptible (I)	Resistant (R)	Not susceptible (I + R)
Penicillin G (*S* ≤ 0.06, *R* > 1.0)	49 (39.5)	74 (59.7)	1 (0.8)	75 (60.5)
Ceftriaxone (*S* ≤ 0.125, *R* > 0.125)	123 (99.2)	0	1 (0.8)	1 (0.8)
Tetracycline (*S* ≤ 0.5, *R* > 1.0)	93 (75.0)	10 (8.1)	21 (16.9)	31 (25.0)
Spectinomycin (*S* ≤ 64, *R* > 64)	124 (100)	0	0	0
Azithromycin (*S* ≤ 0.25, *R* > 0.5)	118 (95.2)	4 (3.2)	2 (1.6)	6 (4.8)
Ciprofloxacin (*S* ≤ 0.03, *R* > 0.06)	73 (58.9)	1 (0.8)	50 (40.3)	51 (41.1)

^a^ according to EUCAST (www.eucast.org)

### Detection of drug resistance mutations via low-density oligonucleotide microarray analysis

The *N. gonorrhoeae* target DNA loci responsible for resistance to beta-lactams, tetracyclines and fluoroquinolones were analysed via hybridisation on a low-density oligonucleotide hydrogel microarray (biochip). The procedure consisted of two steps: (1) a multiplex PCR of the *penA*, *ponA*, *rpsJ*, *gyrA* and *parC* gene segments and (2) hybridisation of fluorescently labelled single-stranded PCR products.

A previously described procedure was used to design the sequences of primers for amplification and oligonucleotides for immobilisation on the biochip following their synthesis and purification [22]. The multiplex PCR procedure is described in Additional file 1 and includes Table S1 with the sequences of the primers.

**Fig. 1:**
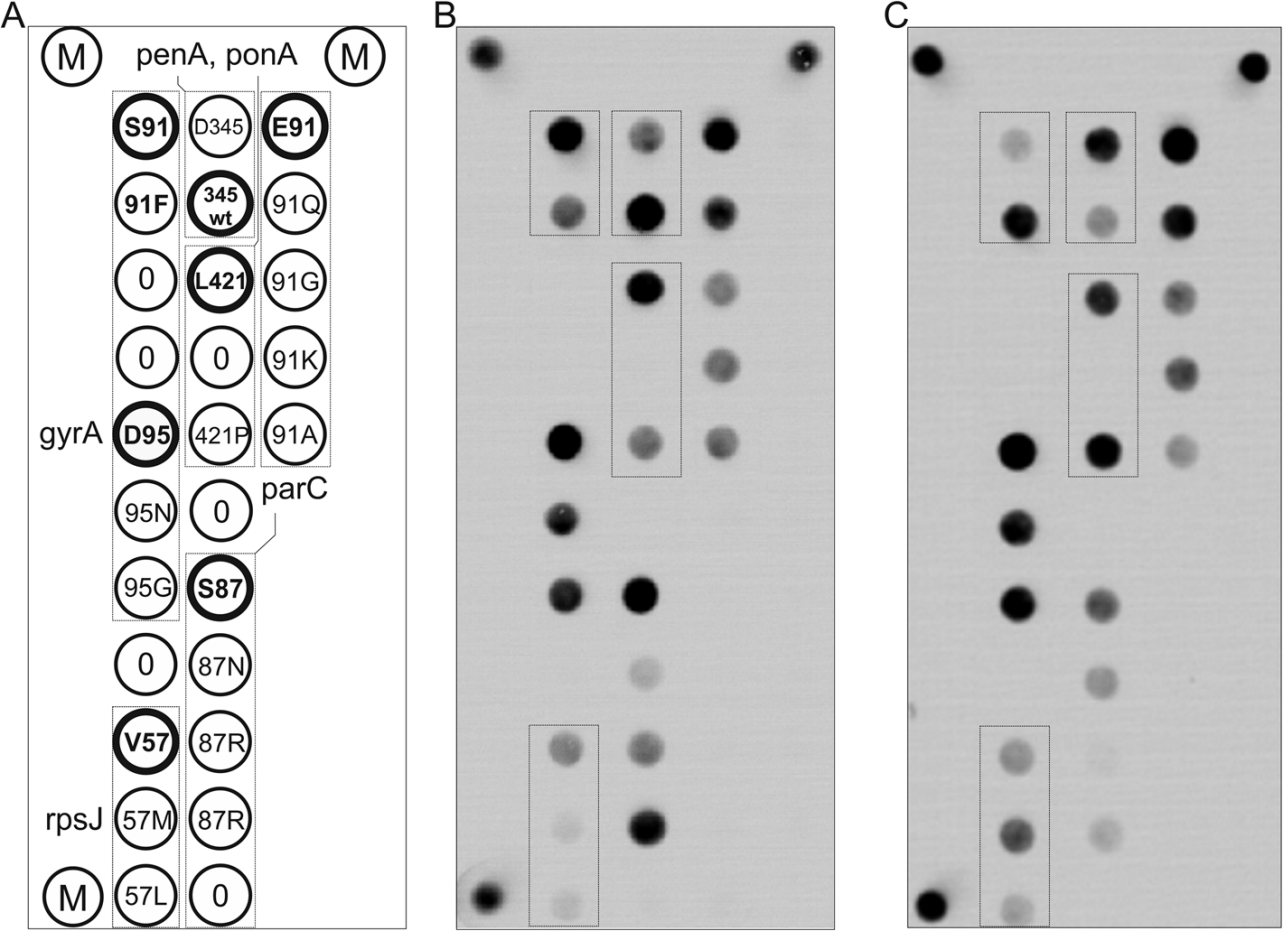
Analysis of *N. gonorrhoeae* drug resistance mutations via hybridisation on a microarray. **a** Hybridisation microarray configuration. Immobilised oligonucleotide probes targeted the insertion of D345 in the *penA* gene, L421P mutation in the *ponA* gene, 2 mutations (V57M and V57L) in the *rpsJ* gene, S91F, D95N, D95G mutations in the *gyrA* gene, and 7 mutations (S87N, S87R, S87R2, E91Q, E91G, E91K and E91A) in the *parC* gene. Elements with wild-type-sequence oligonucleotide probes are depicted with bold circles. M—marker elements with fluorescent label, 0—reference gel elements without oligonucleotides. **b** Hybridisation analysis of the wild-type *N. gonorrhoeae* DNA sample. **c** Hybridisation of a DNA sample containing the following mutations: D345 insertion (*penA*), L421P (*ponA*), V57M (*rpsJ*), and S91F (*gyrA*). Groups with detected mutations are depicted as boxes

The specialised biochip design consisted of 21 elements with immobilised oligonucleotides (Additional file 2: Table S2), 3 marker cells for image acquisition with processing software, and 6 elements of the empty gel used to calculate the background fluorescence intensity (Fig. 1a). Gel elements with immobilised oligonucleotides were arranged in groups, each corresponding to an amplified fragment of genomic DNA. In each group, one element contained an oligonucleotide with the wild-type sequence, and the other elements contained oligonucleotides bearing known mutations at the same position (within 2–5 bp). Perfect or imperfect hybridisation duplexes were formed in the biochip elements depending on the nature of the DNA tested.

The procedures for PCR product hybridisation on the biochip and the processing hybridisation pattern were performed as previously described [23], facilitating the sequence determination of specific analysed sites by comparing fluorescence signals from immobilised oligonucleotide probes for each group. The hybridisation patterns corresponding to wild-type and multiple drug resistance mutations in *N. gonorrhoeae* are shown in Fig. 1b and c, respectively.

## Results

### Molecular typing of *Neisseria gonorrhoeae* isolates

The study was performed with 124 *N. gonorrhoeae* isolates submitted from 9 regions of the Russian Federation over a 12-month period in 2015. These strains (one isolate per patient) were collected from the genital specimens of 92 (74.2 %) men and 32 (25.8 %) women and then identified as *N. gonorrhoeae* based on the presentation of typical colonies on selective agar media, visualisation of Gram-negative diplococci via microscopy, biochemical activity and MALDI-TOF MS spectra.

The sequencing procedure with bacterial DNA extracts revealed 52 *porB* and 27 *tbpB* allele numbers. All these sequences were checked via the online NG-MAST database, and 22 *porB* and 7 *tbpB* nucleotide sequences that were described for the first time were uploaded for new allele number assignation. The most frequently observed sequences included *porB* [5619 (*n* = 14), 37 (*n* = 13), 738 (*n* = 7), and 7325 new (*n* = 6)] and *tbpB* [27 (*n* = 30), 4 (*n* = 20), and 6 (*n* = 20)].

According to the NG-MAST protocol for both the *porB* and *tbpB* alleles, a total of 58 sequence types (STs) were identified and checked via the online database. The prevalence of previously unknown STs was determined, and 35 novel ST numbers (12444–12451, 12524, 12528–12532, 12539, 12540, 12555–12560, 12599–12603, 13054–13061) were obtained from the administrator section of the NG-MAST website. Among the new STs, the majority emerged as combinations of known and novel *porB* and *tbpB* alleles, whereas four STs were absolutely unique: 12447 (*porB* 7287 allele and *tbpB*2098 allele), 12451 (*porB* 7290 allele and *tbpB* 2098 allele), 13059 (*porB* 7590 allele and *tbpB* 2256 allele), and 13060 (*porB* 7591 allele and *tbpB* 2257 allele).

Additionally, 23 clusters (two or more *N. gonorrhoeae* isolates with the same NG-MAST) were identified, whereas 35 STs were represented by single strains. The most frequently observed types (represented by 5 or more isolates) were previously described [9476 (*n* = 14), 807 (*n* = 10), 1544 (*n* = 6), 5714 (*n* = 6) and 1993 (*n* = 5)], whereas most of the new STs were represented in 1–2 isolates, and only 12531 reached 6 strains.

**Fig. 2:**
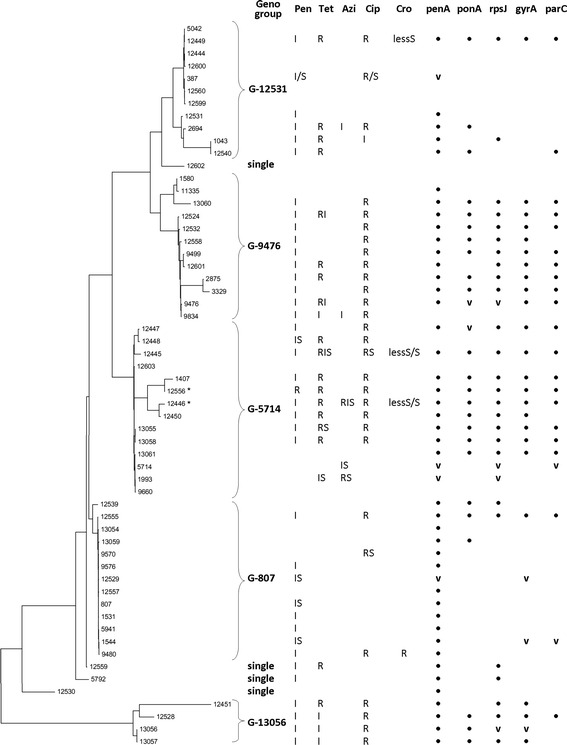
Dendrogram showing isolate similarity in *porB* and *tbpB* alleles (using the maximum likelihood test), sequence types and antimicrobial susceptibility associations. Designations: gaps in the columns indicate the wild-type/susceptible strain; R—resistance; I—intermediate susceptibility; S—susceptibility; RIS, RS, RI, IS—variable susceptibility of strains in the NG-MAST cluster; lessS—decreased susceptibility; points with ‘•’ indicate mutations in the *penA*, *ponA*, *rpsJ*, *gyrA* and *parC* genes (v—variable into NG—MAST cluster); *—multi-drug-resistant sequence types

Further data analysis of *porB* and *tbpB* allele homology (the maximum likelihood test [24]) showed the phylogenetic relationships between certain STs, clustered into ‘genogroups’, comprising the closely related NG-MAST types. Genogroups (G) were named after the most frequently occurring ST within the group (e.g., in G-9476, 52 % were ST 9476, including 11 other STs and consisting of a total of 27 strains). Four major genogroups, G-12531 (11 STs, 19 strains), G-9476 (12 STs, 27 strains), G-5714 (14 STs, 30 strains), G-807 (13 STs, 37 strains), and the small G-13056 genogroup, which includes 4 new STs and was representative of 5 strains, were defined (Fig. 2), encompassing 118 (95.2 %) of the total 124 isolates tested.

### Antimicrobial susceptibility of *N. gonorrhoeae* isolates

The results of the antimicrobial susceptibility testing of all 124 isolates are summarised in Table 1. Of the total isolates, I + R status to penicillin G was more common in *N. gonorrhoeae*; of which 59.7 % (74 of 124) demonstrated intermediate susceptibility and 0.8 % (1 of 124) were resistant strains. Furthermore, high resistance was detected against ciprofloxacin (40.3 %; 50 of 124), whereas only 1 isolate (0.8 %) showed intermediate susceptibility to this antimicrobial. The overall number of tetracycline-resistant *N. gonorrhoeae* isolates was 21 (16.9 %), which was accompanied by 10 (8.1 %) strains of intermediate susceptibility. Among the 124 tested isolates, 2 (1.6 %) were resistant and 4 (3.2 %) demonstrated intermediate susceptibility to azithromycin. Finally, 1 *N. gonorrhoeae* strain only was resistant (MIC = 0.25 mg/L) to ceftriaxone. Because usual N. gonorrhoeae sensitivity to this antibiotic is still quite high: 87 % (108 of 124) strains characterized by the MIC values 0.002–0.008 mg/L, we described additionally some isolates with reduced sensitivity (MIC = 0.06 mg/L) as indicators of developing resistance to ceftriaxone. No isolates resistant to spectinomycin were identified.

Of the total *N. gonorrhoeae* isolates, 2 (1.6 %) multi-drug resistance strains was defined as strictly resistant to 3 or more antimicrobials according to EUCAST criteria, while other 26 (21.0 %) strains were not susceptible (I + R) to 3–4 antimicrobials. One third of all the studied strains (42 of 124; 33.9 %) were susceptible to all tested antimicrobials.

### Antimicrobial resistance genetic determinants of *N. gonorrhoeae* isolates

Data obtained from the molecular typing, antimicrobial susceptibility testing and biochip analysis for each strain are listed in Additional file 3: Table S3.

The chromosomally mediated determinant most frequently detected was D345 in the *penA* gene, which encodes PBP2 and confers resistance to beta-lactams. This mutation appeared in 98 of 124 (79.0 %) *N. gonorrhoeae* strains, whereas the L421P mutation in the *ponA* gene, which also affects susceptibility to beta-lactams, was revealed to be twice as rare (47 strains; 37.9 %). Forty-four V57M and six V57L mutations in the *rpsJ* gene, responsible for resistance to tetracyclines, were detected in 50 (40.3 %) isolates. Similar frequencies were shown for S91F (47 strains; 37.9 %) and D95G (42 strains; 33.9 %) in *gyrA* gene, which is involved in resistance to fluoroquinolones (i.e., ciprofloxacin), whereas both mutations were present in 40 strains (32.3 %). On the other hand, the D95N mutation in the *gyrA* gene was rare and appeared in only 2 strains (in both cases in association with S91F). The S87R mutation in the *parC* gene, which is also involved in resistance to fluoroquinolones, was found in 42 (33.9 %) isolates; this mutation is most typically combined with mutations in the *gyrA* gene. In contrast, E91G was found in only 2 isolates, and the S87N, E91Q, E91K, and E91A mutations were absent.

### Sequence type and antimicrobial resistance associations

A subsequent analysis showed relationships between the antimicrobial susceptibility results and certain sequence types in the described *N. gonorrhoeae* genogroups (Fig. 2).

The G-12531 genogroup was not associated with high-level antimicrobial resistance and was characterised by a low frequency of all revealed genetic determinants. Most typically, isolates from this genogroup (i.e., the ST 12531 cluster) carried a D345 (*penA*) mutation that led to intermediate susceptibility to penicillin G. The most resistant member in this genogroup was an ST 12449 single strain, which showed intermediate susceptibility to penicillin G (MIC = 0.5 mg/L) and signs of decreased susceptibility to ceftriaxone (MIC = 0.06 mg/L), as well as resistance to tetracycline (MIC = 2.0 mg/L) and ciprofloxacin (MIC = 16 mg/L). This strain carried the majority of the revealed mutations, specifically D345 (*penA*), L421P (*ponA*), V57M (*rpsJ*), S91F and D95G (*gyrA*), and S87R (*parC*), which were rare in their neighbours’ STs.

The members of G-9476 genogroup (with the exception of STs 1580 and 11335) showed intermediate susceptibility to penicillin G, high resistance to ciprofloxacin, and, in some NG-MAST types, resistance or intermediate susceptibility to tetracycline that may be associated with high rate of corresponding mutations. The most frequently occurring ST cluster, 9476, with 14 isolates, was variable in terms of its antimicrobial resistance spectra: 11 strains shared D345 (*penA*), L421P (*ponA*), V57M (*rpsJ*), S91F and D95G (*gyrA*), and S87R (*parC*) mutations, whereas 2 strains had no V57M and 1 had no L421P determinants. Notably, the same variability appeared in some other clusters within other genogroups.

The most numerous ST 5714 cluster and the neighbouring ST1993 cluster in the G-5714 genogroup were variables in susceptibility to antimicrobials and drug resistance determinant contents; however, 15 isolates of 9 related STs carried numerous mutations and showed high-level drug resistance. First of all, this refers to the ST 1407-related sequence types, including the STs 12556, 12446 and 12450. The ST 1407 was previously evaluated as an internationally spread MDR *N. gonorrhoeae* clone [9, 14, 25], and in this study, one carried D345 (*penA*), L421P (*ponA*), V57M (*rpsJ*), S91F and D95G (*gyrA*), and S87R (*parC*) mutations, which correlated with reported data [7] and led to intermediate susceptibility to penicillin G and resistance to tetracycline and ciprofloxacin. Moreover, 2 strains in STs 12446 and 12556—closely related to ST 1407, which inherited these mutations—exhibited MDR. Additionally, one isolate in the ST12446 cluster showed decreased susceptibility to ceftriaxone (MIC = 0.06 mg/L).

G-807 had the most STs that were susceptible to antimicrobials, and the frequency of drug resistance determinants was very low in this large genogroup. Only the D345 (*penA*) mutation was preliminarily detected (36 of 37 isolates) to have intermediate susceptibility to penicillin G in most STs. All G-807 genogroup isolates were susceptible to tetracycline and azithromycin, and 3 only STs were resistant to ciprofloxacin (i.e., ST 12555 due to the S91F, D95G and S87R mutations). Moreover, G-807 had a single ST 9480 strain with resistance to ceftriaxone (MIC = 0.25 mg/L), accompanied by intermediate susceptibility to penicillin G and ciprofloxacin.

The G-13056 genogroup was small (4 STs of 5 isolates) but unique and consisted only of the first described STs. The D345 (*penA*), L421P (*ponA*), V57M (*rpsJ*) and S91F (*gyrA*) determinants were typical for the G-13056 members, whereas the rare mutations D95N (*gyrA*) and E91G (*parC*) were revealed in ST 12528. Moreover, all members of this unique genogroup carried porB mutation for amino acids 101 (Q → N) and 102 (R → Y) which may be additive for drug resistance phenomena. As a result, all examples of G-13056 genogroup were ciprofloxacin-resistant and not susceptible (I or R) to penicillin G and tetracycline.

### Distribution of NG-MAST sequence types in participating regions of the Russian Federation and regional variability in *N. gonorrhoeae* antimicrobial resistance

**Fig. 3:**
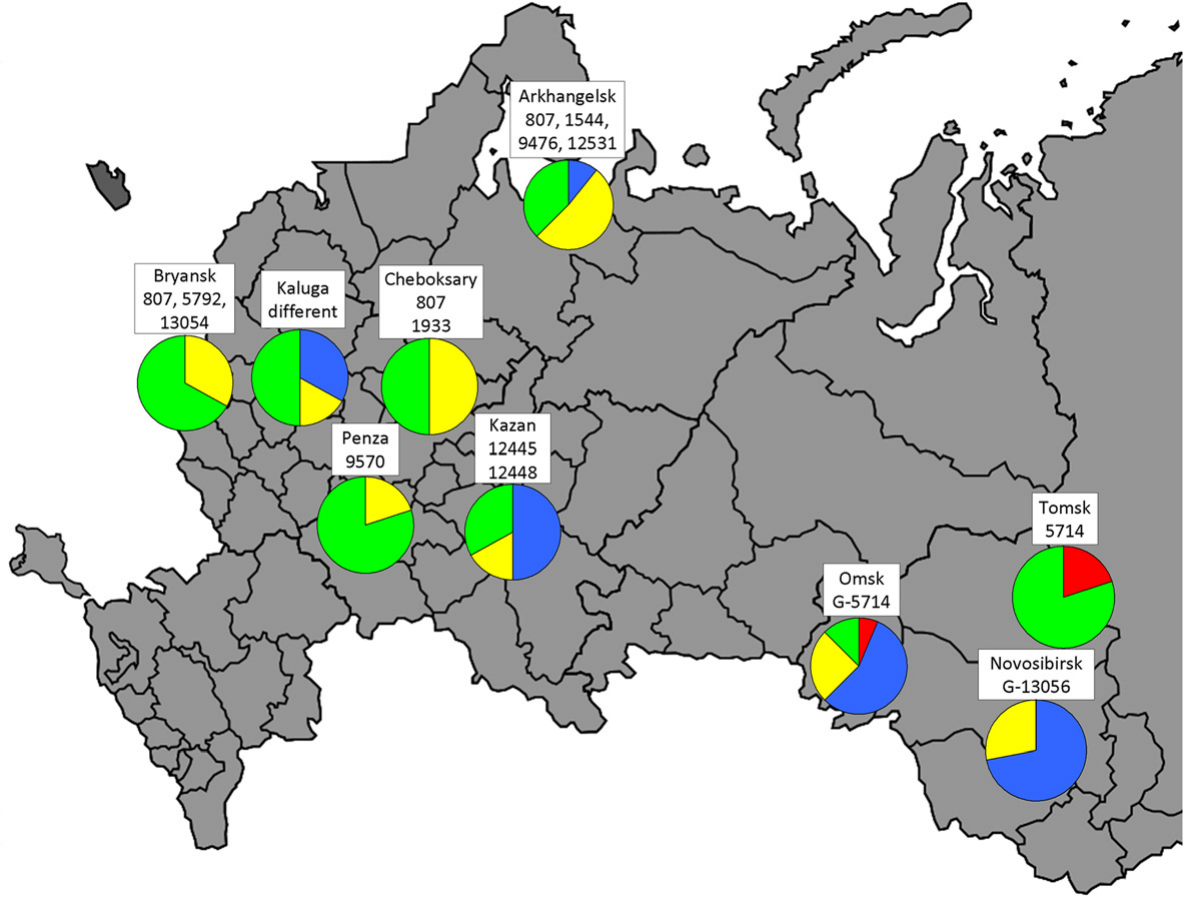
Most frequently observed STs or genogroups in each participating region and antimicrobial resistance in the *N. gonorrhoeae* populations. Designations: green sectors—susceptible isolates (%); yellow sectors—strains not susceptible (I + R) to 1–2 antimicrobials (%); blue sectors—strains not susceptible (I + R) to 3 or more antimicrobials (%); red sectors—multi-drug-resistant isolates (%). The map of Russia was taken from https://commons.wikimedia.org/wiki/File:Blank_Map_-_RussiaFederalSubjects_2007-07.svg source

Only 3 STs (807, 1993 and 5714) were widespread in at least three of the participating regions, whereas considerable diversity in *N. gonorrhoeae* STs was observed between regions, and the predominant types or genogroups were detected in each region. Figure 3 shows the most frequently occurring STs for participating regions that were accompanied by significant variations in antimicrobial susceptibility in *N. gonorrhoeae* populations.

The most closely related samples were obtained from the Penza region, in which only susceptible ST 9570 was found. A low level of *N. gonorrhoeae* antimicrobial resistance was also detected in the Bryansk region, in which three types (STs 807, 5792 and new 13054) were predominant. The Cheboksary region had the common STs 807, 1993 and other *N. gonorrhoeae* isolates, half of which were sensitive to all tested antimicrobials. In the Arkchangelsk region, the STs 807, 1544, 9476 and 12531 were predominant, of which 10.9 % of the strains were not susceptible (I + R) to three or more antimicrobials but 37.5 % were completely susceptible. Moreover, 1 strain resistant to ceftriaxone was revealed in the Arkchangelsk region. The highest variety of STs was identified in the Kaluga region, in which each strain was represented by an individual sequence type and 33 % of the isolates were not susceptible to several antimicrobials, including the 1407 sequence type. In the Kazan region, many isolates were closely related, and the most frequently occurring were the first-described STs 12445 and 12448; therefore, the proportion of non-susceptible isolates was significant (50 %). Multi-drug-resistant *N. gonorrhoeae* isolates are absent in the European part of Russia.

The *N. gonorrhoeae* isolates from the Siberian part of Russia were very different from those in the regions described above. In the Tomsk region, the susceptible G-5714 genogroup was prevalent; whereas the ST 12556-related MDR isolate was also identified in this region. The *N. gonorrhoeae* isolates from the Novosibirsk region were closely genetically linked, and the majority of the strains were related to the unique G-13056 genogroup. In turn, the prevalence of the G-5714 sequence types was observed in the Omsk region, in which the ST 12446 was more common. Due to the high frequencies of antibiotic resistance, genetic determinants in these genogroups and certain STs, the isolates that were not susceptible (I + R) to three or more antimicrobials were widespread in the Omsk and Novosibirsk regions: 56.3 and 71.4 %, respectively (Fig. 3). Moreover, 1 MDR strain and 3 isolates with decreased susceptibility to ceftriaxone were revealed in the Omsk region.

The obtained results indicate *N. gonorrhoeae* epidemiology in Russia as geographically restricted, where significant variations in the most frequently observed STs and genogroups in each of the participating regions led to differences in antimicrobial resistance for each regional gonococcal population.

## Discussion

The present study examined molecular epidemiological characteristics (NG-MAST), susceptibility to penicillin G, ceftriaxone, tetracycline, spectinomycin, azithromycin, and ciprofloxacin, and a spectrum of genetic determinants within the *penA*, *ponA*, *rpsJ*, *gyrA*, and *parC* loci in *N. gonorrhoeae* strains isolated in 9 regions of Russia in 2015.

The NG-MAST analysis showed a diversified population of *N. gonorrhoeae* in Russia with 58 different sequence types among the 124 examined gonococcal isolates, 23 of which were represented by two or more strains; and only 6 STs (807, 1544, 1993, 5714, 9476 and 12531) were represented by five or more isolates. The STs 807, 1993 and 5714 have been widespread in Russia since 2011–2012 [4]; in the present study, these sequence types were still spread throughout at least three participating regions, indicating large sexual transmission chains. Interestingly, ST807 and ST1993 were revealed as predominant clusters in Belorussia [5] and were detected in Kazakhstan [26], which shows the spread of some *N. gonorrhoeae* clones in the former Soviet Union states. However, STs typical for EU counties were rare in the present study, e.g., the internationally spread multi-drug-resistant gonococcal clone ST1407 [9, 14, 25] was established in only one isolate. Finally, most STs evolved locally in Russia, and predominant *N. gonorrhoeae* molecular types were shown in most participating regions. Nevertheless, further data analysis of the homology of the *porB* and *tbpB* alleles established 5 related ‘genogroups’, which demonstrates the molecular phylogeny of *N. gonorrhoeae* population in Russia.

Divisions into genogroups enabled a more robust analysis and exploration of the associations between sequence type and antimicrobial resistance in *N. gonorrhoeae*. The G-12531 and G-807 genogroups were characterised by a low distribution of antimicrobial resistance genetic determinants and included few drug-resistant gonococcal isolates. In contrast, the G-9476 and G-5714 genogroups demonstrated numerous determinants, and the closely related NG-MAST sequence types were found to possess highly similar patterns of drug resistance. The most significant observations included the ST 1407-related first-described STs 12446, 12450 and 12556, which shared numerous mutations that together conferred multi-drug-resistance. Surprisingly, in some cases (STs 1933, 5714, 9476), the isolates of one NG-MAST cluster had variability in terms of their mutation spectra and antimicrobial susceptibility, which was previously shown in *N. gonorrhoeae* isolates from San Francisco, California [27]. This result confirmed that although NG-MAST data may provide valuable information regarding a particular isolate’s antimicrobial susceptibilities, this information is by no means definitive.

The phenotypic antimicrobial susceptibility tests showed high *N. gonorrhoeae* resistance or intermediate susceptibility to previously recommended empiric first-line therapy antimicrobials, such as penicillin G (60.5 %), ciprofloxacin (41.1 %), and tetracycline (25 %), which strongly correlated with the current widespread antimicrobial resistance genetic determinants in gonococcal population in Russia.

Drug-resistant gonococcal isolates in Russia were found in previous studies [4, 28]; however, the rates of antimicrobial resistance were significantly higher. This result may indicate that penicillin G, ciprofloxacin and tetracycline have not been used to treat gonorrhoea in many years; however, we did not register a complete recovery of *N. gonorrhoeae* susceptibility to these antimicrobials and revealed the persistence of antimicrobial resistance genetic determinants without antibiotic selection pressure.

Resistance or intermediate susceptibility to azithromycin was relatively low (4.8 %) in the present study; previously (in 2011–2012), azithromycin resistance was observed in *N. gonorrhoeae* isolates in Russia more higher (16.5–17 %) [4], which provided an opportunity to return to azithromycin for the treatment of gonococcal infections in clinical cases with laboratory-proven *N. gonorrhoeae* susceptibility.

Spectinomycin-resistant *N. gonorrhoeae* related to 32 different STs were detected in some regions of Russia in our previous studies [29, 30]. The present study did not find spectinomycin-resistant or intermediate susceptibility gonococcal isolates; consequently, this antimicrobial is currently listed for the alternative treatment of gonorrhoea in Russia when the patient suffers from a severe beta-lactam allergy.

Using the European EUCAST breakpoint, only 1 (0.8 %) *N. gonorrhoeae* strain was resistant to ceftriaxone (MIC > 0.125 mg/L), and 3 (2.4 %) showed decreased susceptibility (MIC = 0.06 mg/L) to this antimicrobial in 2015. Thus, the decreasing susceptibility to the “last-line” third generation cephalosporins observed in EU countries [6, 7, 9] remains rare in Russia, and we have not reported high-level resistance to ceftriaxone leading to clinical treatment failure. Based on the previously reported [4, 28–31] and current data, ceftriaxone should be recommended for first-line empiric antimicrobial monotherapy for gonorrhoea in Russia, which is in line with the European [31] treatment guidelines. However, the transboundary migration of *N. gonorrhoeae* epidemic strains (ST1407 foremost) and the appearance of new ceftriaxone-resistant strains [6] warrants the proper use and close control of cephalosporins to optimise efficacy.

## Conclusions

This study of the *N. gonorrhoeae* population in the Russian Federation showed predominant resistance or intermediate susceptibility to previously recommended antimicrobials, such as penicillin G, ciprofloxacin and tetracycline. Based on these data, ceftriaxone and spectinomycin are recommended for first-line empirical antimicrobial monotherapy for gonorrhoea. The continuation of the RU-GASP monitoring programme is important for controlling the spread of drug-resistant *N. gonorrhoeae* isolates in Russia.

## Abbreviations

EUCAST, European Committee on Antimicrobial Susceptibility Testing; G, genogroup; MIC, minimum inhibitory concentration; MDR, multi-drug resistance; NG-MAST, *N. gonorrhoeae* Multi-Antigen Sequence Typing; RU-GASP, the Russian Gonococcal Antimicrobial Surveillance Programme; ST, sequence type

## Additional files

Additional file 1: Description of a multiplex PCR procedure for the simultaneous amplification of the *penA*, *ponA*, *rpsJ*, *gyrA* and *parC* gene segments. Table S1. Primers used for the amplification of the *penA*, *ponA*, *rpsJ*, *gyrA* and *parC* fragments. (DOCX 15 kb) Additional file 2: Table S2. List of oligonucleotide probes immobilised in biochip elements for the identification of mutations in the *penA*, *ponA*, *rpsJ*, *gyrA* and *parC* genomic loci. (DOCX 17 kb) Additional file 3: Table S3. List of all isolates used in this study with their sequence types, drug resistance profiles and biochip analysis data. (XLSX 21 kb)
