# Supplementary material for: Molecular epidemiology of drug-resistant Neisseria gonorrhoeae in Russia (Current Status, 2015)
Source: BMC Infect Dis. 2016 Aug 9;16:389. doi: 10.1186/s12879-016-1688-7 (PMC4977856; doi:10.1186/s12879-016-1688-7)
Supplement: Additional file 1: — Description of a multiplex PCR procedure for the simultaneous amplification of the penA, ponA, rpsJ, gyrA and parC gene segments. Table S1. Primers used for the amplification of the penA, ponA, rpsJ, gyrA and parC fragments. (DOCX 15 kb) [file 12879_2016_1688_MOESM1_ESM.docx]

**Multiplex PCR of the *penA, ponA, gyrA*, *parC*, and *rpsJ* gene segments**

Target samples of DNA from *N. gonorrhoeae* were prepared by two-stage multiplex PCR. The lengths of the fragments were 175 bp (*gyrA*), 159 bp (*parC*), 220 bp (*rpsJ*), 228 bp (*penA*) and 201 bp (*ponA*). Primers used for amplification of these segments are listed in Table S1. In the first stage the 30 μl reaction contained 3 μl Hot Start Taq DNA polymerase 10x buffer (SibEnzyme Ltd, Russia), 0.2 mM each dNTP, 2 units of Hot Start Taq DNA polymerase (SibEnzyme), 17 μM of each primer, and 1 μl DNA sample. The cycling conditions were as follows: denaturation at 95°C for 3 min; 25 cycles of denaturation at 95°C for 30 s, annealing at 65°C for 30 s and extension at 72°C for 30 s and a final extension step at 72°C for 5 min.

In the second stage the 33 μl reaction contained 3.3 μl Hot Start Taq DNA polymerase 10x buffer (SibEnzyme), 0.2 mM each dNTP, 8 μM fluorescently labeled dUTP-ImD#49 (Biochip-IMB, LLC, Russia), 2 units of Hot Start Taq DNA polymerase (SibEnzyme), 5 μM of forward primers, 50 μM of reverse primers (Table 1) and 1 μl of PCR product from the first stage. The difference in the concentrations of forward and reverse primers within each pair meant that each reaction yielded predominantly single-stranded fluorescently labeled product. The cycling conditions were as follows: denaturation at 95°C for 3 min; 31 cycle of denaturation at 95°C for 30 s, annealing at 65°C for 30 s and extension at 72°C for 30 s and a final extension step at 72°C for 5 min

| **Table S1. Primers used for amplification of *gyrA*, *parC*, *rpsJ*, *penA* and *ponA* fragments** | | |
| --- | --- | --- |
| Primer | Sequence 5' to 3' | Length |
| gyrA_f | GCTGAAAAATAACTGGAATGCCGCCTACA | 29 |
| gyrA_r | ATCCGAAGTTGCCCTGTCCGTCTA | 24 |
| parC_f | GCATTTTGTTTGCCATGCGCGATATGG | 27 |
| parC_r | AGCGCAAGGTAAAATCCTGAGCCATG | 26 |
| rpsJ_f | CTGCACAAGAAATCGTTGAAACTGCAAA | 28 |
| rpsJ_r | GCCGGCAAATCCAGCTTCATCAGCG | 25 |
| penA_f | GTTCGTGATTGCGAAGGCATTGGATGC | 27 |
| penA_r | CCGATGCCCAATTCATGATAGAAGTCA | 27 |
| ponA_f | GGCCCGAGCGGTCGATAATGAGAAAAT | 27 |
| ponA_r | CGATTGAATGTTTTGCTGTGAAAATCA | 27 |
