# Supplementary material for: Molecular epidemiology of drug-resistant Neisseria gonorrhoeae in Russia (Current Status, 2015)
Source: BMC Infect Dis. 2016 Aug 9;16:389. doi: 10.1186/s12879-016-1688-7 (PMC4977856; doi:10.1186/s12879-016-1688-7)
Supplement: Additional file 2: Table S2. — List of oligonucleotide probes immobilised in biochip elements for the identification of mutations in the penA, ponA, rpsJ, gyrA and parC genomic loci. (DOCX 17 kb) [file 12879_2016_1688_MOESM2_ESM.docx]

| **Table S2. Oligonucleotides used for the microarray** | | | | | |
| --- | --- | --- | --- | --- | --- |
| Oligonucleotide | Amino acid position | Amino acid substitution | Nucleotide substitution | Sequence 5' to 3' | Length |
| S91 | 91 | Ser(wt) |  | GGCGATTCCGCAGTTTACGA | 20 |
| 91F | 91 | Ser 🡪 Phe | TCC 🡪 TTC | GGCGATTTCGCAGTTTACGA | 20 |
| D95 | 95 | Asp(wt) |  | CGCAGTTTACGACACCATCGTCC | 23 |
| 95N | 95 | Asp 🡪 Asn | GAC 🡪 AAC | CGCAGTTTACAACACCATCGTCC | 23 |
| 95G | 95 | Asp 🡪 Gly | GAC 🡪 GGC | GCAGTTTACGGCACCATCGTC | 21 |
| V57 | 57 | Val(wt) |  | TTCTCCGCACGTGAACAAAAC | 21 |
| 57M | 57 | Val 🡪 Met | GTG 🡪 ATG | TTCTCCGCACATGAACAAAAC | 21 |
| 57L | 57 | Val 🡪 Leu | GTG 🡪 TTG | TTCTCCGCACTTGAACAAAAC | 21 |
| 345 wt | 345 | wt |  | CCGTGCGCGATACCCATGT | 19 |
| D345 | 345 | insAsp | 🡪 GAC | GTGCGCGACGATACCCATG | 19 |
| L421 | 421 | Leu(wt) |  | CCGTTGCTGCAGGGGGC | 17 |
| 421P | 421 | Leu 🡪 Pro | CTG 🡪 CCG | CCGTTGCCGCAGGGGG | 16 |
| S87 | 87 | Ser(wt) |  | GCGACAGTTCCGCCTATGA | 19 |
| 87N | 87 | Ser 🡪 Asn | AGT 🡪 AAT | GCGACAATTCCGCCTATGA | 19 |
| 87R | 87 | Ser 🡪 Arg | AGT 🡪 CGT | GCGACCGTTCCGCCTATGA | 19 |
| 87R2 | 87 | Ser 🡪 Arg | AGT 🡪 AGG | GCGACAGGTCCGCCTATGA | 19 |
| E91 | 91 | Glu(wt) |  | CCTATGAGGCGATGGTGCG | 19 |
| 91Q | 91 | Glu 🡪 Gln | GAG 🡪 CAG | CCTATCAGGCGATGGTGCG | 19 |
| 91G | 91 | Glu 🡪 Gly | GAG 🡪 GGG | CCTATGGGGCGATGGTGCG | 19 |
| 91K | 91 | Glu 🡪 Lys | GAG 🡪 AAG | CCTATAAGGCGATGGTGCG | 19 |
| 91A | 91 | Glu 🡪 Ala | GAG 🡪 GCG | CCTATGCGGCGATGGTGCG | 19 |
